# Mortality and causes of death for people with multiple sclerosis: a Finnish nationwide register study

**DOI:** 10.1007/s00415-025-13112-1

**Published:** 2025-05-02

**Authors:** Katariina Kuutti, Sini M. Laakso, Matias Viitala, Sari Atula, Merja Soilu-Hänninen

**Affiliations:** 1 Department of Neurology, Turku University Hospital, Turku, Finland; 2 Clinical Neurosciences, University of Turku, Turku, Finland; 3 Brain Center, Department of Neurology, Helsinki University Hospital, Helsinki, Finland; 4 University of Helsinki, Translational Immunology Research Program, Helsinki, Finland; 5 StellarQ Ltd, Turku, Finland; 6 Aastat Ltd, Turku, Finland; 7 Clinical Neurosciences, University of Helsinki, Helsinki, Finland

**Keywords:** Multiple sclerosis, Mortality, Cause of death, Population-based, Register study

## Abstract

**Introduction:**

Population-based longitudinal data on mortality and causes of death (COD) for people with Multiple Sclerosis (pwMS) is scarce. We studied all-cause and cause-specific mortality in Finnish pwMS in a nationwide registry study.

**Methods:**

PwMS from 1st January 1971 until end of 2019 were identified from the Finnish MS registry and national health care register. Standardized mortality ratios (SMRs), excess death rates (EDRs), life expectancies, and causes of death (COD) were determined by linkage to national registries.

**Results:**

For 16,602 pwMS, 3936 deaths occurred between 1980 and 2020. During 1980–1999, SMR for pwMS was 3.07 (95% CI 2.91–3.25) and EDR 14.05 (95% CI 13.72–14.37), and during 2000–2020 2.18 (95% CI 2.10–2.26) and 7.48 (95% CI 7.2–7.75), respectively. SMRs were higher for female pwMS and for patients diagnosed under age 30. EDRs were higher for males. Risk of death was lower for pwMS diagnosed 1996–2005 versus 1980–1995 (HR 0.49; 95% CI 0.43–0.55; *p* < 0.001). MS was the underlying cause in 51.2%, and a mentioned cause in 73.1% of deaths during 2000–2020. Mortality by underlying cause was higher than expected for gastrointestinal diseases (SMR 2.15, 95% CI 1.53–2.77), respiratory infections (SMR 1.99, 95% CI 1.22–2.75), and vascular diseases (SMR 1.38, 95% CI 1.25–1.51). Median lifetime expectancy was shortened by 7 years.

**Conclusion:**

Excess mortality in Finnish pwMS has decreased during the last 40 years. Life expectancy is shortened by 7 years and MS itself is the most frequent underlying COD. Risk of death is lower for pwMS diagnosed during the therapeutic era.

## Introduction

Multiple sclerosis (MS) is an inflammatory disease of the central nervous system (CNS) characterized by inflammation, demyelination, and axonal loss. Although significant advances in MS treatment have been accomplished, it remains one of the most common causes of neurological disability in young adults globally. MS is associated with functional loss [1], early unemployment [2, 3], and early death; on average, patients with MS (pwMS) have shortened life expectancies by 7–14 years [4–10]. The specific reasons for this premature death remain unclear.

During recent decades, MS epidemiology and treatment have changed in ways that could affect mortality. Incidence has increased [11, 12] and numerous new disease-modifying therapies (DMTs) emerged. With increased use of MRI, developing diagnostic criteria, and improved access to neurological health service, the additional detection of benign cases could result in increasing survival, as well as improved care, rehabilitation, and treatment of symptoms and comorbidities [5, 12, 13]. At the same time, the lifespan of the general population has increased. The changes in MS mortality over time, and how these changes compare to trends in mortality in the general population are incompletely understood.

Standardized mortality ratio (SMR) serves as a measure of relative mortality risk. A meta-analysis by Smyrke et al., covering a period from 1949 to 2013, found no support of a reduced all-cause SMR for pwMS compared to counter-parts over the last 65 years [14]. Accordingly, mortality has decreased among pwMS at similar rates to the general population, possibly due to advances in modern medicine and lifestyle improvements. Although this is consistent with a previous systematic review by Manouchrinia et al., including 12 studies covering the period 1949–2012 [15], it contrasts with several single-population-based studies from the Nordic countries. A rise in survival in MS over time has been observed in Norway [5, 6], in Denmark, [12] and in Sweden [16]. A study on pwMS in New Zealand also found that MS survival had increased relative to the general population by over 15 years [17].

A previous study from Finland showed threefold higher mortality in pwMS diagnosed 1964–1993 in comparison to controls [10]. MS was the underlying cause of death in most of the patients, while infections, gastrointestinal causes, and suicides also contributed to excess mortality. The purpose of this study was to perform an updated investigation at a national level data on all-cause and cause-specific mortality among Finnish pwMS, as well as the changes in mortality rates over time. Comparison was made to national aggregated population data from Statistics Finland.

## Methods

### Study cohort

**Fig. 1:**
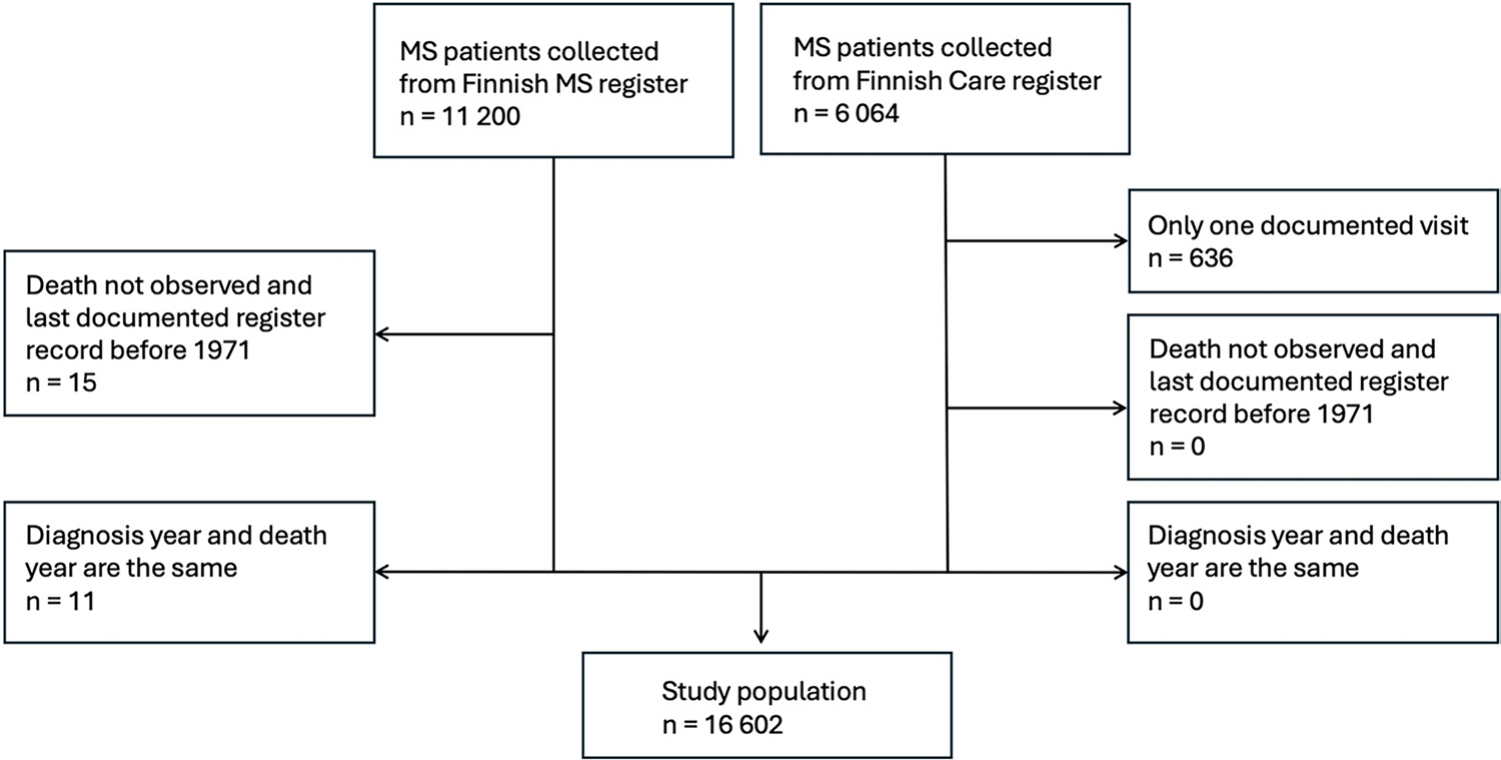
Forming the study population. PwMS were identified and included from both MS-register (prioritized) and national care register (complementing search) starting from the beginning of the year 1971. The following exclusion criteria were used: only one documented visit (*n* = 636 for the national care register data), death not observed and last documented record before the year 1971 (*n* = 15 for MS-register data, *n* = 0 for care register data), and diagnosis year and year of death the same (*n* = 11 for MS-register data, *n* = 0 for care register data). The study population was thus 16 602 pwMS

For this nationwide register-based cohort study, pwMS alive since 1 st January 1971 until the end of 2019 were identified from the Finnish MS-register and the nationwide hospital discharge register HILMO, which started data collection in 1969 (Fig. 1). HILMO includes data from Hospital Discharge Register during 1969–1993, Care Register for Health Care during 1994–2020, and Register for Primary Health Care Visits during 2011–2021. The Finnish MS registry was launched in January 2014. It is integrated with all the largest hospitals’ electronic patient record systems and with both prospective and retrospective data collection covers now data of up to 90% of Finnish pwMS [18]. Deaths and causes of death were collected since 1 st January 1971 until 31 st December 2020 from Statistics Finland and Population Register Centre's Population Information System. Findata approval (permit number THL/623/14.02.00/2021) for the study was obtained, and the data were stored and analyzed within the data secure environment of the Wellbeing County of Southwest Finland, Atolli.

### Ethical approval

According to Finnish law, ethics committee approval was not required, since the study was based on administrative register data and did not involve any contact with patients. The study was approved by the Turku and Helsinki University Hospital Research Services. The data processing practices followed the EU Data Protection Directive rules.

### Standardized mortality ratio and excess death rate

Yearly Standardized mortality ratios (SMR) were determined by dividing the number of observed deaths among pwMS by the number of expected deaths in the general population matched by sex, age group, and calendar-year. For longer time periods, Poisson regression was used for SMR calculation. Excess death rate (EDR) was determined by the difference between the observed number of deaths per 1000 person-years and the corresponding expected number. For the data on general population, we used national aggregated population data from Statistics Finland. To avoid bias caused by sharp MS incidence and prevalence changes, we analyzed SMR for 20-year periods of follow-up and used additionally EDR that is considered by some authors a better parameter for longitudinal mortality analyses [6].

Causes of death were grouped similar to what was used in [19] and cause-specific SMRs (CS-SMR’s) are presented for the latter observation period of 2000–2020. CS-SMRs were calculated using Opensource Statistics Finland yearly death count data for sex and each ICD-10 code.

### Charlson comorbidity index (CCI)

To assess for the effect of comorbidities on mortality in pwMS, we calculated the CCI as described previously [20]. To analyze the effect of comorbidities on mortality, we compared the CCI for pwMS deceased during 2000–2020 to those alive at the end of 2020.

### Life expectancy and survival from diagnosis

Median life expectancies were calculated for both observed data and compared to the general population. Life tables provided by Human Mortality Database were utilized to calculate expected life expectancies. Also, survival from date of MS diagnosis was assessed fitting survival curves since corresponding diagnosis date to death or end of follow-up.

### Statistical analyses

Partial dates for MS-register data were imputed as middle of the year or month, where applicable. Descriptive analyses were conducted using summary statistics. Numerical variables were expressed as means with standard deviations (SDs) or medians with interquartile ranges (IQRs). Categorical variables were expressed as frequencies and proportions based on non-missing data.

15-year survival for sex, diagnosis age, and diagnosis year was analyzed fitting Proportional Hazards Regression model using Efron approximation for ties. If death was not observed, patients were censored at 15-year mark or at final day of 2020, which occurred first. Median observed life expectancies were calculated fitting survival curves since birth until death or final day of 2020. Median expected life expectancies were calculated utilizing sex and birth year matched life tables where expected survival was estimated using Ederer method. Survival from diagnosis was analyzed based on survival curves since date of diagnosis to death or final day of 2020. Survival analyses were limited for diagnosis years between 1975 and 2019.

Standardized mortality ratios were calculated using yearly death rate data for 5-year age groups, sex, and calendar-year. SMRs for longer time periods were estimated using Poisson regression, where number of observed deaths was modeled with logarithm-transformed expected deaths as offset. EDR per 1000 person-years was calculated utilizing difference between observed and expected death, and corresponding population at risk. Confidence intervals for EDRs were calculated using normal approximation.

*P* values under 0.05 were considered as significant. RStudio (Version 2023.06.01) was used for all data modification and analysis.

## Results

In our study population of 16 602 pwMS, median age at MS onset was 36.0 years (Q1–Q3 28.0–46.0) and majority (69.7%) had been diagnosed after 1995 (Table 1). For this study population, a total of 4124 deaths were recorded starting from the year 1971. The range of the follow-up was 1 to 45 years. Forming the study population is demonstrated in Fig. 1.

**Table 1:** Demographics of the study cohort, number of deaths recorded, age at death, and Charlson Comorbidity Index for all pwMS identified in the study

	Total population *n* = 16,602	Females *n* = 11,324 (68.2%)	Males *n* = 5278 (31.8%)
Age at MS onset*; median (Q1-Q3)	36.0 (28.0–46.0)	35.0 (27.0–45.0)	37.0 (28.0–47.0)
Age at MS diagnosis; median (Q1-Q3)	39.0 (30.0–48.0)	38.0 (30.0–48.0)	39.0 (31.0–49.0)
*Diagnosis year; n* *(%)*			
Before 1975	1062 (6.4%)	626 (5.5%)	436 (8.3%)
1975–1985	1842 (11.1%)	1137 (10.0%)	705 (13.4%)
1986–1995	2122 (12.8%)	1433 (12.7%)	689 (13.1%)
1996–2005	4716 (28.4%)	3319 (29.3%)	1397 (26.5%)
2006–2019	6860 (41.3%)	4809 (42.5%)	2051 (38.9%)
*Number of deaths*			
Before 1980	188	80	108
1980–1989	511	260	251
1990–1999	739	429	310
2000–2009	1048	615	433
2010–2020	1638	1030	608
*Observed Charlson Comorbidity Index (CCI); median (Q1–Q3)*			
Died between 2000 and 2020	1.00 (0.00–3.00)	1.00 (0.00–3.00)	1.00 (0.00–3.00)
Alive at end of 2020	0.00 (0.00–1.00)	0.00 (0.00–1.00)	0.00 (0.00–1.00)

^*^Missing MS onset dates imputed based on 10-year diagnosis interval groups

Overall mortality between 1980 and 2020 was higher for pwMS compared to the general population, with an SMR of 2.40 (95% CI 2.32, 2.47; *p* < 0.001) for all patients and 2.65 (95% CI 2.55, 2.76; *p* < 0.001) for females (Table 2). During the whole study period of 1980–2020, EDR was 9.03 (95% CI 8.74, 9.32). During 1980–1999, SMR for pwMS was 3.07 (95% CI 2.91, 3.25; *p* < 0.001) and EDR 14.05 (95% CI 13.72–14.37), and during 2000–2020 2.18 (95% CI 2.10–2.26) and 7.48 (95% CI 7.2–7.75), respectively. SMR was higher for female pwMS and EDR for male patients at both periods (Table 2).

**Table 2:** Mortality for pwMS calculated by SMR and EDR based on overall mortality during 1980–2020 for females and males, and 15-year mortality from diagnosis by both diagnosis year and age at diagnosis

	Observed	Expected	SMR (95% CI)	EDR (95% CI)
*Overall mortality 1980–2020*				
All patients	3936	1640	***2.40 (2.32, 2.47)	9.03 (8.74, 9.32)
Females	2334	880	***2.65 (2.55, 2.76)	8.26 (7.99, 8.52)
Males	1602	760	***2.10 (2.00, 2.21)	10.76 (10.42, 11.10)
*Overall mortality 1980–1999*				
All patients	1250	407	***3.07 (2.90, 3.24)	14.05 (13.72, 14.37)
Females	689	194	***3.55 (3.30, 3.83)	12.67 (12.37, 12.96)
Males	561	213	***2.62 (2.42, 2.85)	16.63 (16.25, 17.01)
*Overall mortality 2000–2020*				
All patients	2686	1233	***2.18 (2.10, 2.26)	7.48 (7.20, 7.75)
Females	1645	686	***2.40 (2.28, 2.52)	7.00 (6.74, 7.26)
Males	1041	547	***1.90 (1.79, 2.02)	8.61 (8.29, 8.94)
*15-year mortality 1975–2020 by diagnosis year*				
1975–1985	479	149	***3.19 (2.91, 3.49)	13.36 (13.05, 13.68)
1986–1995	373	137	***2.71 (2.45, 3.00)	7.98 (7.72, 8.24)
1996–2005	492	251	***1.96 (1.79, 2.14)	3.56 (3.35, 3.76)
*15-year mortality 1975–2020 by age at diagnosis*				
Under 30y	114	24	***4.76 (3.96, 5.72)	3.13 (2.99, 3.26)
30–39y	245	66	***3.70 (3.27, 4.20)	4.79 (4.61, 4.97)
40–49y	336	126	***2.66 (2.39, 2.96)	6.49 (6.25, 6.72)
Equal to or over 50y	649	321	***2.01 (1.86, 2.17)	13.90 (13.50, 14.30)

*SMR* Standardized mortality ratio, *EDR* excess death rate; y year

^***^p < 0.001

^**^p < 0.01

^*^p < 0.05

**Fig. 2:**
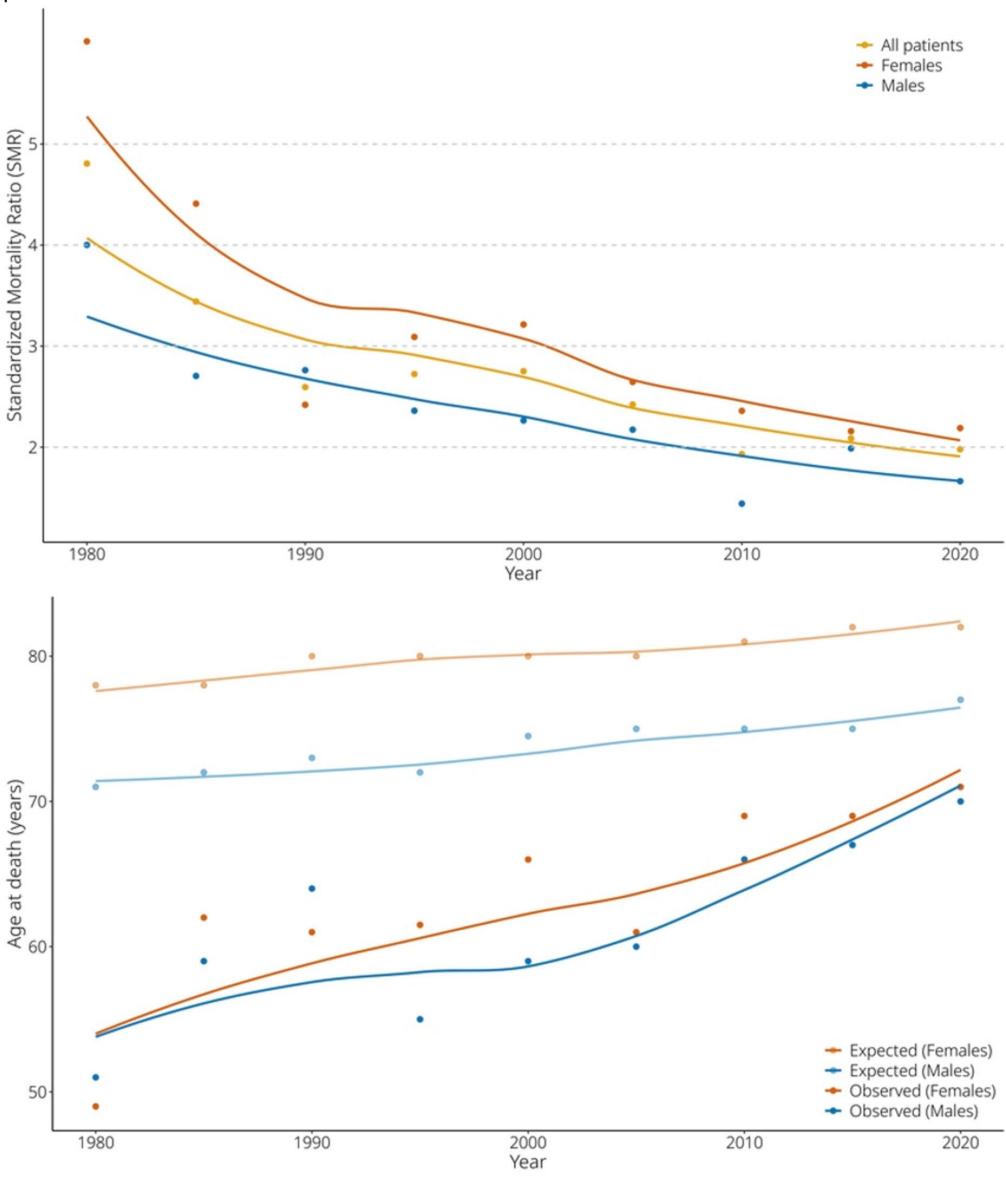
**A** Changes in SMR and **B** changes in age at death from 1980 to 2020 shown for female and male pwMS. For age at death, observed age at death is compared to population-based expected age for females (orange) and males (blue) separately

Comorbidities were more common for pwMS deceased between 2000 and 2020 than for those alive at the end of 2020 (CCI median 1.00, Q1–Q3 0.00–3.00 vs 0.00, Q1–Q3 0.00–1.00, respectively; *p* < 0.001). Overall, we saw a steady decline in SMR and increase in the age at death for both female and male pwMS from 1980 to 2020 (Fig. 2). Age at death steadily increased from a median of 52.0 years (Q1-Q3 43.5–60.0) before 1980, to 69.0 years (Q1–Q3 60.0–77.0) during 2010–2020, and similar findings were made for both female and male pwMS (Table 1, Fig. 2). Population-based expected ages at death and ages at death of the pwMS in 10-year intervals are shown in Table 3.

**Table 3:** Median life expectancies in comparison to the general population and median survival from diagnosis for female and male pwMS

	MS patients	General population
Median life expectancy (95% CI)		
All patients	77.3 (76.8, 78.1)	84.0
Female	79.0 (78.5, 79.5)	85.7
Male	74.7 (73.8, 75.5)	79.0
Median survival since MS diagnosis (95% CI)		
All patients	34.4 (33.3, 35.2)	
Female	36.3 (34.9, 37.6)	
Male	31.1 (29.2, 32.2)	

	Median age at death (IQR)	
	Observed	Expected
All patients		
1980–1989	56.0 (46.0–65.0)	77.0 (72.0–79.0)
1990–1999	60.0 (50.0–70.0)	78.0 (73.0–80.0)
2000–2009	62.0 (54.0–73.0)	79.0 (75.0–81.0)
2010–2020	69.0 (60.0–77.0)	80.0 (77.0–82.0)
Female		
1980–1989	57.5 (46.0–66.0)	78.0 (77.0–80.0)
1990–1999	61.0 (50.0–72.0)	80.0 (78.0–81.0)
2000–2009	63.0 (54.0–75.0)	80.0 (79.0–82.0)
2010–2020	69.0 (61.0–77.0)	82.0 (80.0–84.0)
Male		
1980–1989	56.0 (46.0–65.0)	72.0 (70.0–74.0)
1990–1999	58.0 (48.0–68.0)	72.0 (71.0–74.0)
2000–2009	61.0 (53.0–70.0)	74.0 (72.0–76.0)
2010–2020	68.0 (59.0–76.0)	75.5 (72.0–79.0)

To control the follow-up time, we assessed 15-year mortality from diagnosis (Table 2). There was a clear decreasing trend of mortality for pwMS diagnosed between 1975 and 1985 vs those diagnosed between 1996 and 2005, with SMR decreasing from 3.19 (95% CI 2.91, 3.49) to 1.96 (1.79, 2.14), respectively. When assessed by age at diagnosis, SMR for 15-year mortality from diagnosis for pwMS diagnosed before the age of 30 years was 4.76 (95% CI 3.96, 5.72), whereas for those diagnosed after the age of 50 years, SMR was 2.01 (95% CI 1.86, 2.17).

Using life tables, we analyzed the effect of MS on life expectancy in comparison to the general population. In female pwMS, median survival was 79.0 years (95% CI 78.5, 79.5) in comparison to expected 84 years and in male pwMS 74.7 years (95% CI 73.8, 75.5) in comparison to expected 79 years. Median survival from diagnosis was 36.3 years (95% CI 34.9, 37.6) in females and 31.1 (95% CI 29.2, 32.2) in males (Table 3).

We then calculated hazard ratios (HR) for death according to demographic variables for pwMS. Male pwMS had a higher HR compared to females, 1.82 (95% CI 1.64, 2.00; *p* < 0.001). MS diagnosis after 1995 was a protective factor from death, HR 0.55 (95% CI 0.48, 0.63) compared to earlier time intervals of diagnosis (*p* < 0.001). Older patients had obviously a higher overall HR of death compared to patients diagnosed under the age of 30 years, HR 8.58 (95% CI 7.09, 10.38; *p* < 0.001) (Table 4).

**Table 4:** Hazard ratio (HR) for death according to demographic variables for pwMS

Sex	HR (95% CI)	*p* value
Females	Ref	
Males	1.82 (1.64, 2.00)	< 0.001
Diagnosis year		
1975–1985	1.51 (1.32, 1.74)	< 0.001
1986–1995	Ref	
1996–2005	0.55 (0.48, 0.63)	< 0.001
Diagnosis age		
Under 30 y	Ref	
30–39 y	1.66 (1.34, 2.06)	< 0.001
40–49 y	2.85 (2.32, 3.50)	< 0.001
Equal to or over 50 y	8.58 (7.09, 10.38)	< 0.001

^*^y year

For the COD analysis, we collected data on COD separately during 1980–1999 and 2000–2020, and grouped CODs based on ICD-10 codes (Table 5). For pwMS, underlying, contributing and immediate COD data were available. Comparative data of the general Finnish population were available only for underlying COD. MS was the underlying COD in 59.1% of pwMS during 1980–1999 vs 51.3% during 2000–2020, and either underlying or contributing COD in 73.9% vs 73.5% of the patients, respectively. After MS itself, vascular diseases and cancer were the two most common underlying or contributing CODs in pwMS during both time periods. The most common immediate cause of death for pwMS was respiratory infection (23.6% during 1980–1999 and 28.0% during 2000–2020).

**Table 5:** Underlying, contributing COD and immediate COD in pwMS 1980–1999 and 2000–2020

	Died between 1980 and 1999 (*n* = 1250)			Died between 2000 and 2020 (*n* = 2686)		
Cause of death (COD)	Underlying	Underlying or contributing	Immediate	Underlying	Underlying or contributing	Immediate
Multiple sclerosis	739 (59.1%)	924 (73.9%)	0	1376 (51.2%)	1973 (73.5%)	0
Other neurologic	19 (1.5%)	42 (3.4%)	< 5	118 (4.4%)	232 (8.6%)	22 (0.8%)
Vascular disease	197 (15.8%)	251 (20.1%)	62 (5.0%)	424 (15.8%)	748 (27.8%)	178 (6.6%)
Venous thrombosis and embolism	< 5	< 5	< 5	5 (0.2%)	8 (0.3%)	0
Chronic respiratory disease	16 (1.3%)	24 (1.9%)	6 (0.5%)	29 (1.1%)	97 (3.6%)	22 (0.8%)
Respiratory infection	56 (4.5%)	71 (5.7%)	295 (23.6%)	26 (1.0%)	119 (4.4%)	753 (28.0%)
Aspiration pneumonia	0	< 5	35 (2.8%)	0	10 (0.4%)	87 (3.2%)
Liver disease	5 (0.4%)	10 (0.8%)	0	49 (1.8%)	77 (2.9%)	5 (0.2%)
Gastrointestinal tract	19 (1.5%)	27 (2.2%)	17 (1.4%)	46 (1.7%)	91 (3.4%)	28 (1.0%)
Chronic kidney and bladder disease	13 (1.0%)	31 (2.5%)	5 (0.4%)	6 (0.2%)	61 (2.3%)	17 (0.6%)
Urinary tract infection	5 (0.4%)	26 (2.1%)	16 (1.3%)	8 (0.3%)	45 (1.7%)	47 (1.7%)
Cancer (all types)	89 (7.1%)	98 (7.8%)	0	375 (14.0%)	440 (16.4%)	14 (0.5%)
Other infection	5 (0.4%)	9 (0.7%)	< 5	9 (0.3%)	23 (0.9%)	21 (0.8%)
Sepsis	< 5	< 5	36 (2.9%)	8 (0.3%)	16 (0.6%)	78 (2.9%)
Musculoskeletal	< 5	5 (0.4%)	0	6 (0.2%)	29 (1.1%)	< 5
Pregnancy and childbirth	0	0	0	0	0	0
Congenital disease	0	< 5	0	< 5	9 (0.3%)	0
Disease of eyes or ears	0	< 5	0	0	< 5	0
Skin disease	0	7 (0.6%)	0	< 5	32 (1.2%)	7 (0.3%)
Blood disease	0	< 5	0	< 5	24 (0.9%)	< 5
Psychiatric disease	< 5	20 (1.6%)	0	5 (0.2%)	93 (3.5%)	0
Dementia	< 5	8 (0.6%)	0	28 (1.0%)	55 (2.0%)	0
Metabolic and endocrine	5 (0.4%)	37 (3.0%)	< 5	19 (0.7%)	189 (7.0%)	< 5
Complications of medical care	0	< 5	0	0	5 (0.2%)	5 (0.2%)
Suicide	17 (1.4%)	17 (1.4%)	0	46 (1.7%)	47 (1.7%)	0
Accident	19 (1.5%)	21 (1.7%)	12 (1.0%)	73 (2.7%)	93 (3.5%)	10 (0.4%)
Homicide	< 5	< 5	0	< 5	< 5	0
Other	35 (2.8%)	44 (3.5%)	9 (0.7%)	16 (0.6%)	23 (0.9%)	12 (0.4%)

Causes identified in < 5 patients are not reported in detail

Mortality by underlying cause (Cause specific SMR, CS-SMR; Table 6) during 2000–2020 was higher than expected for diseases of the gastrointestinal tract (SMR 2.15, 95% CI 1.61, 2.87; *p* < 0.001), respiratory infections (SMR 1.99, 95% CI 1.35, 2.92; *p* < 0.001), and for vascular diseases (SMR 1.38, 95% CI 1.26, 1.52; *p* < 0.001) (Table 6).

**Table 6:** CS-SMRs in pwMS compared to the general population 2000–2020

	Observed	Expected	CS-SMR (95% CI)
Vascular disease	424	307	***1.38 (1.26, 1.52)
Cancer (all types)	375	372	1.01 (0.91, 1.11)
Other neurologic	118	109	1.09 (0.91, 1.30)
Liver disease	49	58	0.85 (0.64, 1.13)
Gastrointestinal tract	46	21	***2.15 (1.61, 2.87)
Chronic respiratory disease	29	37	0.79 (0.55, 1.13)
Dementia	28	28	1.01 (0.70, 1.46)
Respiratory infection	26	13	***1.99 (1.35, 2.92)
Metabolic and endocrine	19	19	1.00 (0.64, 1.56)

CS-SMR refers to underlying causes of death

^***^p < 0.001

^**^p < 0.01

^*^p < 0.05

## Discussion

In this population-based nationwide study on mortality and causes of death in Finnish pwMS with up to 45 years of follow-up, we showed decreasing mortality from threefold to twofold and steadily increasing age at death through time. Median life expectancy was shortened by 7 years. Female pwMS survived a median of 36 and male pwMS a median of 31 years from diagnosis. Risk of death was lowest for the pwMS without comorbidities and diagnosis during the DMT era.

The overall SMR of 2.4 and EDR of 9 for pwMS in our cohort who died during 1980–2020 is in line with several previous studies [6, 14, 15]. In the previous national mortality study from Finland for pwMS diagnosed between 1964 and 1993, a threefold SMR was reported [10]. In our cohort of pwMS who died between 1980 and 1999, SMR was similarly 3.07. For patients who died between 2000 and 2020, the overall SMR had decreased to 2.14 and EDR halved from 14 to 7. Median life expectancies, years of life lost in comparison to the general population, and survival from diagnosis were similar or close to previous studies from other Nordic countries, and a recent study from the western Finland [6, 12, 23]. As a caveat in our study, we did not have the onset dates for the older part of our study cohort retrieved from the national care register HILMO, and thence had to use year of diagnosis instead of disease onset in the analyses.

Risk of death was significantly lower in pwMS diagnosed between 1996 and 2005 vs 1980 and 1995. Long-term studies evaluating benefit of DMTs are few and the data on effects of treatment on survival in MS are limited. Our later diagnosis period of 1995–2005 coincides with the availability of DMT’s for MS in Finland. Interferons were available first since mid-1990 s, and interferons and glatiramer acetate were the most used DMT’s until 2013 [21]. Our results indirectly suggest that lower hazard of death in our later observation period could be attributed to the availability of DMTs. Our results are in line with a previous registry-based study from western Norway, which showed that treatment-eligible pwMS diagnosed in the DMT era had the lowest risk of mortality [6].

A recent study using multiple administrative health databases from four Canadian provinces studied directly the association between the first-generation and second-generation DMTs and all-cause mortality in pwMS, followed from 1996 to 2017. They showed that earlier DMT initiation (betainterferon or glatiramer acetate vs no exposure) was associated with a significant effect on mortality, while later initiation was not. However, the survival advantage with earlier initiation diminished over time, no longer reaching statistical significance at 15 years [22]. In a recent study from Finland, concerning a regional cohort of pwMS diagnosed in 1971–2010 and followed up until the end of the year 2019, the use of betainterferon or glatiramer acetate vs no exposure was also shown to be associated with better survival, while the decade of diagnosis did not have an impact on survival [23]. In our study cohort, we did not have data of DMT use in the patients from the care register HILMO, and thence could not analyze the DMT impact on survival directly.

Alternatively, decreasing mortality in pwMS during our later observation period could be attributed to improvements in the general healthcare of the chronically disabled, such as developments in rehabilitation, symptomatic therapies and care for comorbidities, as well as diagnosis of more benign cases with increased diagnostic sensitivity. A population-based study from Denmark, spanning 6 decades, indicated that the decrease in MS mortality began well beyond the DMT era, before use of MRI became widespread, and before the McDonald diagnostic criteria were introduced [12]. They speculated that a change in the MS cohorts with fewer malignant cases may be a significant contributor. This may also be a contributing factor in our study, since we could see a decreasing SMR and increasing age at death already since 1980 s. Shorter follow-up time for the participants included toward the end of the study period also offer a plausible explanation for the improved survival.

When assessing all-cause mortality by the age at MS diagnosis, SMR was highest for the youngest diagnosis group, and lowest for the oldest diagnosis group (4.76 for those diagnosed at under 30 years vs 2.01 for those diagnosed at over 50 years). This observation was similar to previous studies from Norway and Sweden [6, 16] and plausible as the mortality in the younger background population is low and other mortality risks increase with age. The overall excess mortality in our study was higher for females than males (SMR 2.65 vs 2.1, respectively). A previous Canadian study found a similar sex difference in mortality [24] and as in our study, but no direct explanation for this difference could be found. Speculations have included the higher incidence of MS in females, some unknown environmental factors or a difference in comorbidities, but none of these have been established. Males and those diagnosed at a higher age, however, had higher hazard ratios for 15-year mortality due to the shorter remaining life expectancy of older age groups and males. In line with this, EDRs were higher in male pwMS in our study. The opposite trends between genders in these two measures of mortality have also been found in the studies from Norway and Denmark [6, 12]. It is considered to reflect the difference between these measures, such that EDR more directly than SMR reflects the difference in the number of lives lost [12].

Similarly, as in the previous Finnish study and several other studies [6, 25, 26], MS was the underlying or contributing COD in the majority of the pwMS also in the current study. Mortality by underlying cause in pwMS during 2000–2020 was higher than expected for diseases of the gastrointestinal tract (SMR 2.15, 95% CI 1.53, 2.77), respiratory infections (SMR 1.99, 95% CI 1.22, 2.75), and for vascular diseases (SMR 1.38, 95% CI 1.25, 1.51). After MS itself, vascular diseases and cancer were leading causes of death, but cancer mortality was not higher than in the general population like vascular mortality was. Infections and gastrointestinal causes were identified as causes of the excess mortality in Finnish pwMS also in the previous national study in Finland [10], and cardiovascular causes in a more recent study from Southwest Finland [27]. Suicide risk has been reported to be increased in pwMS [7], and in the previous Finnish study the SMR for suicide was 1.7 [10]. Suicide data were not available from the open data source that was used for comparison with general population in this study, and thence, SMR for suicide could not be calculated. It was the underlying or contributing cause of death in 1.7% of the pwMS in this study, a similar proportion as in a previous Swedish hospital-based study [28].

It has previously been shown that pwMS have an increased risk of developing multiple comorbidities, and comorbidities are associated with diagnostic delays and increased mortality [29, 30]. In this study, we analyzed the impact of comorbidities on mortality using CCI as a measure of comorbidity burden. CCI predicts 10-year survival in patients with multiple comorbidities. Comorbidities were more common for pwMS deceased between 2000 and 2020 than for those alive at the end of 2020, indicating that comorbidities increased the risk of death also in our cohort. In a previous study from Finland, we observed a survival disadvantage within MS associated with comorbidity for circulatory diseases [27]. Cardiovascular diseases were among the three most common COD contributing to excess mortality also in this study. This emphasizes the importance of identification and good care of comorbidities to improve prognosis of our patients.

## Strengths and limitations

The strength of our study is a large nationwide population-based cohort of over 16 000 pwMS followed up to 45 years. Limitations of the study arise from the lack of a specified control group from the general population. This limits the comparisons, such that only the underlying causes of death could be compared to the general population. Also, analysis of the effect of comorbidities was limited to pwMS, since no data of comorbidities were available for the general population. Impact of DMTs on mortality was indirectly concluded from the year of diagnosis, since DMT data were not available for patients identified from the national care register instead of the MS registry. Dates of diagnosis in the care register may have been registered with delay and were not confirmed by chart review similarly as the MS registry data is. High efficacy DMTs have only been available since last 20 years, and it is too early days to analyze their impact on mortality in a lifelong disease diagnosed at young age.

It is important to note that calculating the median age at death annually poses a limitation in the more recent diagnostic cohorts, such that many individuals may still be live at the end of the follow-up. Consequently, the observed deaths may be disproportionately from individuals with more aggressive disease courses or higher mortality risk, while individuals who have reached older ages are less likely included given the shorter observation window. Therefore, we may have underestimated the true median age at death for the more recent cohorts.

## Conclusion

In summary, our study provides longitudinal population-based survival data of Finnish pwMS, demonstrating a decreasing mortality and lowered risk of death of pwMS diagnosed during the DMT era. While the mortality is still twofold and life expectancy shortened by 7 years, with reduced diagnostic delay, availability of more potent DMTs, more aggressive treatment approaches, and better care of comorbidities, the survival of MS could approach that of the general population. Future studies should address the question whether the use of high-efficacy DMTs affects mortality in pwMS.

## Data Availability

These data are subject to third party restrictions. Permission to access the data may be applied from Findata (https://findata.fi/en/).
